# (2,2′-Bipyridine-6,6′-dicarboxyl­ato-κ^3^ *N*,*N*′,*O* ^6^)(6′-carb­oxy-2,2′-bipyridine-6-carboxyl­ato-κ^3^ *N*,*N*′,*O* ^6^)cobalt(III)

**DOI:** 10.1107/S1600536812009816

**Published:** 2012-03-14

**Authors:** Huimin Wang, Xiaojun Gu, Bingbing Zhang, Haiquan Su

**Affiliations:** a College of Life Sciences, Inner Mongolia University, Hohhot 010021, People’s Republic of China; b School of Chemistry and Chemical Engineering, Inner Mongolia University, Hohhot 010021, People’s Republic of China

## Abstract

The Co^III^ atom in the title compound, [Co(C_12_H_6_N_2_O_4_)(C_12_H_7_N_2_O_4_)], is six-coordinated in a distorted octa­hedral geometry by four N atoms and two O atoms of the chelating 2,2′-bipyridine-6,6′-dicarboxyl­ate and 6′-carb­oxy-2,2′-bipyridine-6-carboxyl­ate ligands. Intermolecular O—H⋯O hydrogen bonds and face-to-face π-stacking inter­actions [centroid–centroid distance = 3.6352 (16) Å] between inversion-related pyridine rings link adjacent mononuclear units into a two-dimensional supra­molecular structure, and several inter­molecular C—H⋯O inter­actions are also observed.

## Related literature

For the structure of a Co^II^ compound with pyridine-2,6-dicarboxyl­ate and 4,4′-bipyridine, see: Ghosh *et al.* (2005 ▶). For the structures and thermal properties of five *Ln* ^III^ (*Ln* is a lanthanide) compounds with the title ligand, see: Wang *et al.* (2010 ▶), for a related Rh^III^ compound with the title ligand, see: Wang *et al.* (2012 ▶) and for a related Ni^II^ compound with the title ligand, see: Wang, Su *et al.* (2009 ▶). For the structures and magnetic properties of [Gd^III^_4_Co^II^Co^III^(μ_3_-OH)_3_(μ_3_-O)(pydc)_6_(H_2_O)_5_]·8H_2_O (pydc = 2,5-pyridinedicarboxylate dianion), see: Wang, Yue *et al.* (2009 ▶).
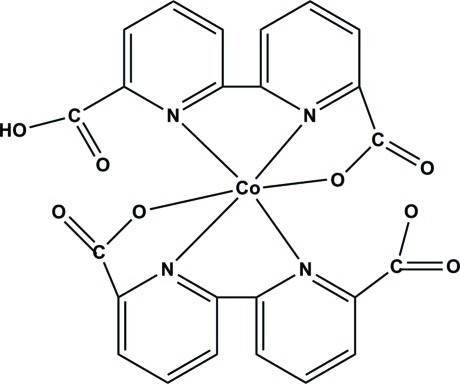


## Experimental

### 

#### Crystal data

[Co(C_12_H_6_N_2_O_4_)(C_12_H_7_N_2_O_4_)]
*M* *_r_* = 544.31
Monoclinic, 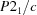

*a* = 9.3329 (19) Å
*b* = 13.561 (3) Å
*c* = 16.894 (3) Å
β = 100.70 (3)°
*V* = 2101.0 (7) Å^3^
*Z* = 4
Mo *K*α radiation
μ = 0.88 mm^−1^
*T* = 153 K
0.26 × 0.20 × 0.08 mm

#### Data collection

Rigaku Saturn CCD area-detector diffractometer
Absorption correction: multi-scan (*CrystalClear*; Rigaku/MSC, 2005 ▶) *T*_min_ = 0.803, *T*_max_ = 0.933
13969 measured reflections
3696 independent reflections
3219 reflections with *I* > 2σ(*I*)
*R*_int_ = 0.043

#### Refinement

*R*[*F* ^2^ > 2σ(*F* ^2^)] = 0.038
*wR*(*F* ^2^) = 0.091
*S* = 1.06
3696 reflections
335 parameters
H-atom parameters constrained
Δρ_max_ = 0.24 e Å^−3^
Δρ_min_ = −0.47 e Å^−3^

### 

Data collection: *CrystalClear* (Rigaku/MSC, 2005 ▶); cell refinement: *CrystalClear*; data reduction: *CrystalClear*; program(s) used to solve structure: *SHELXS97* (Sheldrick, 2008 ▶); program(s) used to refine structure: *SHELXL97* (Sheldrick, 2008 ▶); molecular graphics: *DIAMOND* (Brandenburg & Putz, 2006 ▶); software used to prepare material for publication: *publCIF* (Westrip, 2010 ▶).

## Supplementary Material

Crystal structure: contains datablock(s) I, global. DOI: 10.1107/S1600536812009816/qm2056sup1.cif

Structure factors: contains datablock(s) I. DOI: 10.1107/S1600536812009816/qm2056Isup2.hkl

Additional supplementary materials:  crystallographic information; 3D view; checkCIF report

## Figures and Tables

**Table 1:** Hydrogen-bond geometry (Å, °)

*D*—H⋯*A*	*D*—H	H⋯*A*	*D*⋯*A*	*D*—H⋯*A*
O10—H10*A*⋯O7^i^	0.82	1.65	2.445 (3)	164
C15—H15⋯O2^i^	0.93	2.58	3.391 (3)	147
C8—H8⋯O10^ii^	0.93	2.41	3.142 (3)	135
C16—H16⋯O6^iii^	0.93	2.52	3.044 (3)	116
C20—H20⋯O1^iv^	0.93	2.55	3.315 (3)	140
C22—H22⋯O9^v^	0.93	2.35	3.091 (3)	136

Symmetry codes: (i) 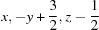; (ii) 
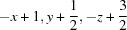
; (iii) 
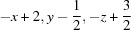
; (iv) 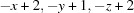; (v) 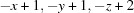.

**Fig. 1.**
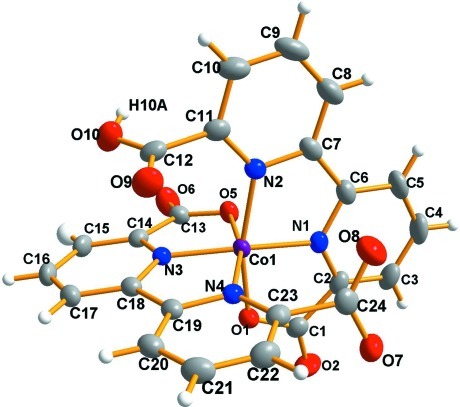
The structure of the title compound with 50% probability displacement ellipsoids.

**Fig. 2.**
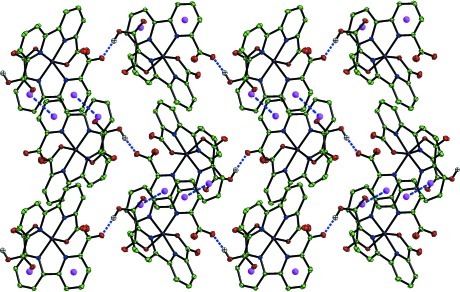
The two-dimensional layer structure of the title compound via hydrogen bonds and face-to-face π-stacking interactions.

## supplementary crystallographic information

### Comment

In recent years, many bipyridine dicarboxylic acid ligands such as 2,2'-dipyridine-4,4'-dicarboxylic acid, 2,2'-dipyridine-5,5'-dicarboxylic acid and 2,2'-dipyridine-6,6'-dicarboxylic acid have been used in metal–organic coordination chemistry because of their diverse coordination modes, which leads to more stable and fascinating architectures (Wang *et al.*, 2012). Some X-ray crystal structures constructed from the title ligand and metal ions, such as [Ni*L*_2_].4H_2_O (Wang, Su *et al.*, 2009), [Ln_3_*L*_4_(*HL*)(H_2_O)_2_].12H_2_O (Ln=Ce, Nd, Pr) (Wang *et al.*, 2010) and [Rh*L*(*HL*)] (Wang *et al.*, 2012), have been investigated previously. In this work, we report the synthesis and structure of the title compound, and a careful literature survey showed that it is the first compound constructed from Co^III^ ion and the title ligand.

In the title compound, the Co^III^ center is six-coordinated in a distorted octahedral geometry by four N atoms and two O atoms of two chelating ligands *L* and *HL* (*H_2_L*= 2,2'-bipyridine-6,6'-dicarboxylic acid) (Fig. 1). Support for the assignment of a +3 oxidation state to Co comes from the Co—N and Co—O bond distances [1.8546 (19) and 1.9941 (19) Å for Co—N bonds and 1.9018 (16) and 1.9055 (18) Å for Co—O bonds which are shorter than those reported for Co^II^ compounds (Ghosh *et al.*, 2005; Wang, Yue *et al.*, 2009). The coordinated bipyridine fragments are nearly coplanar [see torsion angles = 2.0 (3) and 2.2 (3)° in Table 1].

In the structure, the hydrogen-bond donor O10 is connected to the acceptor O7 from adjacent molecule to form a one-dimensional chain along the c-axis (O10—H10A···O7^i^ = 1.65 Å, i = x, -y+3/2, z-1/2, Table 2). Moreover, the adjacent chains are linked into a two-dimensional layer by π–π contacts between inversion-related pyridine rings with Cg7···Cg8ii distance of 3.6352 Å (Fig. 2). Cg7 and Cg8 are the centroids of the pyridine rings (N3, C14–C18) and (N4, C19–C23), respectively (symmetry code: ii = -x, 1-y, -z). Several intermolecular C—H···O interactions contribute to stabilize the crystal structure.

### Experimental

The title compound was obtained by the reaction of the mixture of Co(NO_3_)_2_.6H_2_O, and 2,2'-dipyridine-6,6'-dicarboxylic acid in a molar ratio of 1:0.8 and 8 ml of water under hydrothermal conditions (at 393 K for 4 days and cooled to room temperature with a 2 K h^-1^ rate). The brown block crystals were washed by water (Yield: 30%).

### Refinement

The H atoms were placed in geometrically idealized positions (C—H = 0.95 Å and O—H = 0.82–0.84 Å) with *U*_iso_(H) = 1.2*U*_eq_(C) and *U*_iso_(H) = 1.5*U*_eq_(O).

### Crystal data

**Table:**

[Co(C_12_H_6_N_2_O_4_)(C_12_H_7_N_2_O_4_)]	*Z* = 4
*M**_r_* = 544.31	*F*(000) = 1104
Monoclinic, *P*2_1_/*c*	*D*_x_ = 1.721 Mg m^−^^3^
Hall symbol: -P 2ybc	Mo *K*α radiation, λ = 0.71073 Å
*a* = 9.3329 (19) Å	µ = 0.88 mm^−^^1^
*b* = 13.561 (3) Å	*T* = 153 K
*c* = 16.894 (3) Å	Block, brown
β = 100.70 (3)°	0.26 × 0.20 × 0.08 mm
*V* = 2101.0 (7) Å^3^	

### Data collection

**Table:**

Rigaku Saturn CCD area-detector diffractometer	3696 independent reflections
Radiation source: fine-focus sealed tube	3219 reflections with *I* > 2σ(*I*)
Graphite monochromator	*R*_int_ = 0.043
ω and φ scans	θ_max_ = 25.0°, θ_min_ = 1.9°
Absorption correction: multi-scan (*CrystalClear*; Rigaku/MSC, 2005)	*h* = −11→8
*T*_min_ = 0.803, *T*_max_ = 0.933	*k* = −16→16
13969 measured reflections	*l* = −18→20

### Refinement

**Table:**

Refinement on *F*^2^	Primary atom site location: structure-invariant direct methods
Least-squares matrix: full	Secondary atom site location: difference Fourier map
*R*[*F*^2^ > 2σ(*F*^2^)] = 0.038	Hydrogen site location: inferred from neighbouring sites
*wR*(*F*^2^) = 0.091	H-atom parameters constrained
*S* = 1.06	*w* = 1/[σ^2^(*F*_o_^2^) + (0.0499*P*)^2^ + 0.3348*P*] where *P* = (*F*_o_^2^ + 2*F*_c_^2^)/3
3696 reflections	(Δ/σ)_max_ = 0.001
335 parameters	Δρ_max_ = 0.24 e Å^−^^3^
0 restraints	Δρ_min_ = −0.47 e Å^−^^3^

### Special details

**Table:**

Geometry. All e.s.d.'s (except the e.s.d. in the dihedral angle between two l.s. planes) are estimated using the full covariance matrix. The cell e.s.d.'s are taken into account individually in the estimation of e.s.d.'s in distances, angles and torsion angles; correlations between e.s.d.'s in cell parameters are only used when they are defined by crystal symmetry. An approximate (isotropic) treatment of cell e.s.d.'s is used for estimating e.s.d.'s involving l.s. planes.
Refinement. Refinement of *F*^2^ against ALL reflections. The weighted *R*-factor *wR* and goodness of fit *S* are based on *F*^2^, conventional *R*-factors *R* are based on *F*, with *F* set to zero for negative *F*^2^. The threshold expression of *F*^2^ > σ(*F*^2^) is used only for calculating *R*-factors(gt) *etc*. and is not relevant to the choice of reflections for refinement. *R*-factors based on *F*^2^ are statistically about twice as large as those based on *F*, and *R*- factors based on ALL data will be even larger.

### Fractional atomic coordinates and isotropic or equivalent isotropic displacement parameters (Å^2^)

**Table:**

	*x*	*y*	*z*	*U*_iso_*/*U*_eq_	
Co1	0.84031 (3)	0.73728 (2)	0.932821 (17)	0.02082 (12)	
O1	1.01239 (19)	0.73257 (12)	1.01357 (9)	0.0282 (4)	
O2	1.1381 (2)	0.81870 (16)	1.11603 (10)	0.0460 (5)	
O5	0.93674 (17)	0.81669 (12)	0.86609 (9)	0.0267 (4)	
O6	1.0973 (2)	0.80414 (14)	0.78364 (11)	0.0450 (5)	
N1	0.8057 (2)	0.85278 (14)	0.98537 (11)	0.0251 (4)	
N2	0.6513 (2)	0.77321 (14)	0.86292 (11)	0.0234 (4)	
N3	0.9085 (2)	0.63364 (14)	0.87852 (10)	0.0216 (4)	
N4	0.7596 (2)	0.62602 (14)	0.98808 (10)	0.0234 (4)	
C1	1.0325 (3)	0.80793 (19)	1.06293 (13)	0.0299 (6)	
C2	0.9115 (3)	0.88248 (18)	1.04578 (13)	0.0286 (6)	
C3	0.9052 (3)	0.97481 (19)	1.07909 (14)	0.0365 (6)	
H3	0.9766	0.9956	1.1218	0.044*	
C4	0.7900 (3)	1.0357 (2)	1.04739 (16)	0.0416 (7)	
H4	0.7823	1.0975	1.0702	0.050*	
C5	0.6856 (3)	1.00621 (19)	0.98213 (17)	0.0382 (7)	
H5	0.6106	1.0484	0.9595	0.046*	
C6	0.6962 (3)	0.91155 (18)	0.95143 (14)	0.0291 (6)	
C7	0.6051 (3)	0.86495 (18)	0.88142 (14)	0.0296 (6)	
C8	0.4839 (3)	0.9092 (2)	0.83680 (16)	0.0392 (7)	
H8	0.4554	0.9716	0.8505	0.047*	
C9	0.4047 (3)	0.8600 (2)	0.77141 (17)	0.0421 (7)	
H9	0.3225	0.8888	0.7406	0.051*	
C10	0.4492 (3)	0.7678 (2)	0.75259 (17)	0.0396 (7)	
H10	0.3971	0.7335	0.7089	0.048*	
C11	0.5733 (3)	0.72615 (19)	0.79957 (14)	0.0281 (6)	
C12	0.6111 (3)	0.62148 (19)	0.78089 (13)	0.0286 (6)	
C13	1.0147 (3)	0.76672 (18)	0.82270 (14)	0.0272 (6)	
C14	0.9955 (2)	0.65787 (18)	0.82720 (12)	0.0240 (5)	
C15	1.0604 (3)	0.58537 (19)	0.78857 (13)	0.0302 (6)	
H15	1.1204	0.6014	0.7523	0.036*	
C16	1.0332 (3)	0.48753 (19)	0.80572 (14)	0.0323 (6)	
H16	1.0746	0.4373	0.7800	0.039*	
C17	0.9449 (3)	0.46388 (18)	0.86091 (13)	0.0286 (6)	
H17	0.9271	0.3985	0.8724	0.034*	
C18	0.8844 (3)	0.53988 (17)	0.89816 (13)	0.0232 (5)	
C19	0.7946 (3)	0.53543 (17)	0.96114 (13)	0.0236 (5)	
C20	0.7504 (3)	0.44827 (19)	0.99124 (14)	0.0298 (6)	
H20	0.7748	0.3879	0.9712	0.036*	
C21	0.6691 (3)	0.4523 (2)	1.05193 (15)	0.0351 (6)	
H21	0.6366	0.3947	1.0727	0.042*	
C22	0.6372 (3)	0.5430 (2)	1.08100 (14)	0.0336 (6)	
H22	0.5850	0.5469	1.1227	0.040*	
C23	0.6831 (3)	0.62865 (19)	1.04793 (13)	0.0272 (5)	
C24	0.6458 (3)	0.7264 (2)	1.08152 (15)	0.0319 (6)	
O9	0.5735 (2)	0.55690 (13)	0.82239 (10)	0.0396 (5)	
O7	0.7342 (2)	0.74748 (14)	1.14778 (11)	0.0418 (5)	
O10	0.6766 (2)	0.60709 (13)	0.72153 (10)	0.0401 (5)	
H10A	0.6806	0.6591	0.6972	0.060*	
O8	0.5416 (2)	0.77371 (16)	1.04846 (13)	0.0504 (5)	

### Atomic displacement parameters (Å^2^)

**Table:**

	*U*^11^	*U*^22^	*U*^33^	*U*^12^	*U*^13^	*U*^23^
Co1	0.0249 (2)	0.01599 (19)	0.02186 (19)	0.00158 (13)	0.00513 (13)	0.00048 (12)
O1	0.0310 (10)	0.0244 (9)	0.0281 (9)	0.0023 (7)	0.0025 (7)	−0.0007 (7)
O2	0.0432 (12)	0.0567 (14)	0.0329 (9)	−0.0020 (10)	−0.0064 (9)	−0.0069 (9)
O5	0.0325 (10)	0.0184 (9)	0.0312 (8)	−0.0005 (7)	0.0108 (7)	0.0014 (7)
O6	0.0528 (13)	0.0339 (11)	0.0572 (12)	−0.0022 (9)	0.0331 (10)	0.0083 (9)
N1	0.0325 (12)	0.0199 (11)	0.0246 (9)	0.0003 (9)	0.0097 (9)	−0.0003 (8)
N2	0.0244 (11)	0.0210 (11)	0.0250 (10)	−0.0006 (8)	0.0047 (8)	0.0025 (8)
N3	0.0246 (10)	0.0196 (10)	0.0204 (9)	0.0012 (8)	0.0037 (8)	0.0010 (8)
N4	0.0259 (11)	0.0225 (11)	0.0216 (9)	0.0015 (9)	0.0036 (8)	0.0012 (8)
C1	0.0369 (15)	0.0295 (14)	0.0239 (12)	−0.0025 (12)	0.0076 (11)	0.0032 (10)
C2	0.0384 (15)	0.0260 (13)	0.0239 (12)	−0.0047 (11)	0.0123 (11)	−0.0022 (10)
C3	0.0550 (18)	0.0283 (15)	0.0287 (13)	−0.0095 (13)	0.0143 (12)	−0.0072 (11)
C4	0.0585 (19)	0.0241 (14)	0.0481 (16)	−0.0011 (14)	0.0255 (15)	−0.0100 (12)
C5	0.0461 (17)	0.0222 (14)	0.0509 (16)	0.0084 (12)	0.0211 (14)	0.0005 (12)
C6	0.0319 (14)	0.0241 (13)	0.0345 (13)	0.0029 (11)	0.0143 (11)	0.0010 (11)
C7	0.0301 (14)	0.0255 (14)	0.0354 (13)	0.0058 (11)	0.0118 (11)	0.0073 (11)
C8	0.0365 (16)	0.0324 (16)	0.0497 (16)	0.0108 (13)	0.0103 (13)	0.0089 (13)
C9	0.0304 (15)	0.0444 (18)	0.0502 (16)	0.0071 (13)	0.0041 (13)	0.0218 (14)
C10	0.0317 (16)	0.0448 (18)	0.0384 (15)	−0.0024 (13)	−0.0036 (12)	0.0092 (13)
C11	0.0280 (14)	0.0306 (14)	0.0260 (12)	−0.0044 (11)	0.0057 (11)	0.0071 (10)
C12	0.0307 (14)	0.0304 (14)	0.0222 (12)	−0.0080 (11)	−0.0014 (11)	0.0017 (11)
C13	0.0277 (14)	0.0241 (13)	0.0295 (13)	0.0013 (11)	0.0044 (11)	0.0032 (10)
C14	0.0243 (13)	0.0265 (13)	0.0208 (11)	−0.0005 (10)	0.0032 (10)	0.0004 (10)
C15	0.0344 (15)	0.0339 (15)	0.0241 (12)	0.0030 (12)	0.0099 (11)	−0.0028 (11)
C16	0.0386 (15)	0.0291 (14)	0.0292 (13)	0.0056 (12)	0.0065 (11)	−0.0088 (11)
C17	0.0350 (15)	0.0198 (13)	0.0291 (12)	0.0002 (11)	0.0012 (11)	−0.0022 (10)
C18	0.0240 (13)	0.0201 (12)	0.0236 (11)	−0.0015 (10)	−0.0001 (10)	−0.0001 (9)
C19	0.0245 (13)	0.0206 (12)	0.0241 (11)	−0.0006 (10)	0.0005 (10)	0.0001 (9)
C20	0.0316 (14)	0.0236 (14)	0.0323 (13)	−0.0008 (11)	0.0011 (11)	0.0027 (10)
C21	0.0356 (15)	0.0312 (15)	0.0373 (14)	−0.0088 (12)	0.0040 (12)	0.0105 (12)
C22	0.0339 (15)	0.0387 (16)	0.0299 (13)	−0.0049 (12)	0.0103 (11)	0.0050 (11)
C23	0.0245 (13)	0.0327 (14)	0.0238 (11)	0.0005 (11)	0.0029 (10)	0.0012 (10)
C24	0.0345 (16)	0.0333 (15)	0.0325 (14)	0.0019 (12)	0.0183 (12)	0.0058 (11)
O9	0.0513 (12)	0.0310 (11)	0.0378 (10)	−0.0101 (9)	0.0116 (9)	0.0074 (8)
O7	0.0494 (12)	0.0421 (12)	0.0340 (10)	0.0100 (9)	0.0078 (9)	−0.0072 (8)
O10	0.0612 (13)	0.0283 (10)	0.0332 (10)	−0.0079 (9)	0.0148 (9)	0.0017 (8)
O8	0.0472 (13)	0.0497 (13)	0.0542 (12)	0.0178 (11)	0.0092 (10)	0.0045 (10)

### Geometric parameters (Å, º)

**Table:**

Co1—N3	1.8546 (19)	C8—H8	0.9300
Co1—N1	1.8580 (19)	C9—C10	1.372 (4)
Co1—O5	1.9018 (16)	C9—H9	0.9300
Co1—O1	1.9055 (18)	C10—C11	1.397 (4)
Co1—N2	1.993 (2)	C10—H10	0.9300
Co1—N4	1.9941 (19)	C11—C12	1.510 (4)
O1—C1	1.310 (3)	C12—O9	1.214 (3)
O2—C1	1.212 (3)	C12—O10	1.282 (3)
O5—C13	1.313 (3)	C13—C14	1.491 (3)
O6—C13	1.215 (3)	C14—C15	1.381 (3)
N1—C6	1.338 (3)	C15—C16	1.391 (4)
N1—C2	1.344 (3)	C15—H15	0.9300
N2—C11	1.339 (3)	C16—C17	1.392 (3)
N2—C7	1.372 (3)	C16—H16	0.9300
N3—C14	1.334 (3)	C17—C18	1.382 (3)
N3—C18	1.343 (3)	C17—H17	0.9300
N4—C23	1.342 (3)	C18—C19	1.473 (3)
N4—C19	1.371 (3)	C19—C20	1.380 (3)
C1—C2	1.503 (4)	C20—C21	1.385 (3)
C2—C3	1.378 (3)	C20—H20	0.9300
C3—C4	1.383 (4)	C21—C22	1.377 (4)
C3—H3	0.9300	C21—H21	0.9300
C4—C5	1.388 (4)	C22—C23	1.391 (3)
C4—H4	0.9300	C22—H22	0.9300
C5—C6	1.395 (3)	C23—C24	1.508 (4)
C5—H5	0.9300	C24—O8	1.211 (3)
C6—C7	1.466 (3)	C24—O7	1.293 (3)
C7—C8	1.375 (3)	O10—H10A	0.8200
C8—C9	1.382 (4)		
			
N3—Co1—N1	168.91 (8)	C7—C8—H8	120.3
N3—Co1—O5	83.81 (8)	C9—C8—H8	120.3
N1—Co1—O5	87.09 (8)	C10—C9—C8	119.0 (2)
N3—Co1—O1	90.43 (8)	C10—C9—H9	120.5
N1—Co1—O1	83.36 (8)	C8—C9—H9	120.5
O5—Co1—O1	90.84 (7)	C9—C10—C11	119.5 (3)
N3—Co1—N2	103.87 (8)	C9—C10—H10	120.3
N1—Co1—N2	82.17 (8)	C11—C10—H10	120.3
O5—Co1—N2	88.54 (7)	N2—C11—C10	122.1 (2)
O1—Co1—N2	165.53 (7)	N2—C11—C12	120.1 (2)
N3—Co1—N4	81.51 (8)	C10—C11—C12	117.6 (2)
N1—Co1—N4	107.45 (8)	O9—C12—O10	125.0 (3)
O5—Co1—N4	165.32 (7)	O9—C12—C11	117.0 (2)
O1—Co1—N4	88.91 (7)	O10—C12—C11	118.0 (2)
N2—Co1—N4	95.32 (8)	O6—C13—O5	124.1 (2)
C1—O1—Co1	115.57 (15)	O6—C13—C14	122.4 (2)
C13—O5—Co1	114.27 (15)	O5—C13—C14	113.5 (2)
C6—N1—C2	122.8 (2)	N3—C14—C15	120.3 (2)
C6—N1—Co1	118.95 (16)	N3—C14—C13	111.7 (2)
C2—N1—Co1	116.55 (16)	C15—C14—C13	127.8 (2)
C11—N2—C7	117.8 (2)	C14—C15—C16	117.9 (2)
C11—N2—Co1	130.32 (17)	C14—C15—H15	121.1
C7—N2—Co1	111.60 (15)	C16—C15—H15	121.1
C14—N3—C18	122.9 (2)	C15—C16—C17	120.9 (2)
C14—N3—Co1	116.17 (16)	C15—C16—H16	119.6
C18—N3—Co1	120.42 (15)	C17—C16—H16	119.6
C23—N4—C19	117.8 (2)	C18—C17—C16	118.4 (2)
C23—N4—Co1	129.24 (17)	C18—C17—H17	120.8
C19—N4—Co1	112.87 (15)	C16—C17—H17	120.8
O2—C1—O1	124.4 (2)	N3—C18—C17	119.5 (2)
O2—C1—C2	122.7 (2)	N3—C18—C19	111.17 (19)
O1—C1—C2	112.8 (2)	C17—C18—C19	129.3 (2)
N1—C2—C3	120.1 (2)	N4—C19—C20	122.6 (2)
N1—C2—C1	111.4 (2)	N4—C19—C18	113.95 (19)
C3—C2—C1	128.3 (2)	C20—C19—C18	123.4 (2)
C2—C3—C4	118.3 (2)	C19—C20—C21	118.8 (2)
C2—C3—H3	120.9	C19—C20—H20	120.6
C4—C3—H3	120.9	C21—C20—H20	120.6
C3—C4—C5	121.1 (2)	C22—C21—C20	118.9 (2)
C3—C4—H4	119.5	C22—C21—H21	120.5
C5—C4—H4	119.5	C20—C21—H21	120.5
C4—C5—C6	118.2 (3)	C21—C22—C23	120.0 (2)
C4—C5—H5	120.9	C21—C22—H22	120.0
C6—C5—H5	120.9	C23—C22—H22	120.0
N1—C6—C5	119.3 (2)	N4—C23—C22	121.8 (2)
N1—C6—C7	111.8 (2)	N4—C23—C24	119.9 (2)
C5—C6—C7	128.8 (2)	C22—C23—C24	118.3 (2)
N2—C7—C8	122.1 (2)	O8—C24—O7	127.6 (3)
N2—C7—C6	114.5 (2)	O8—C24—C23	120.9 (2)
C8—C7—C6	123.3 (2)	O7—C24—C23	111.4 (2)
C7—C8—C9	119.4 (3)	C12—O10—H10A	109.5
			
N3—Co1—O1—C1	167.56 (17)	Co1—N1—C6—C7	8.9 (3)
N1—Co1—O1—C1	−3.23 (16)	C4—C5—C6—N1	−0.1 (4)
O5—Co1—O1—C1	83.75 (16)	C4—C5—C6—C7	−176.8 (2)
N2—Co1—O1—C1	−3.7 (4)	C11—N2—C7—C8	−0.7 (3)
N4—Co1—O1—C1	−110.93 (17)	Co1—N2—C7—C8	174.33 (19)
N3—Co1—O5—C13	−6.93 (16)	C11—N2—C7—C6	−180.0 (2)
N1—Co1—O5—C13	166.73 (16)	Co1—N2—C7—C6	−5.0 (2)
O1—Co1—O5—C13	83.42 (16)	N1—C6—C7—N2	−2.0 (3)
N2—Co1—O5—C13	−111.04 (16)	C5—C6—C7—N2	174.9 (2)
N4—Co1—O5—C13	−5.5 (4)	N1—C6—C7—C8	178.8 (2)
N3—Co1—N1—C6	114.4 (4)	C5—C6—C7—C8	−4.4 (4)
O5—Co1—N1—C6	79.54 (18)	N2—C7—C8—C9	0.5 (4)
O1—Co1—N1—C6	170.72 (19)	C6—C7—C8—C9	179.7 (2)
N2—Co1—N1—C6	−9.38 (18)	C7—C8—C9—C10	0.0 (4)
N4—Co1—N1—C6	−102.53 (18)	C8—C9—C10—C11	−0.2 (4)
N3—Co1—N1—C2	−51.4 (5)	C7—N2—C11—C10	0.5 (3)
O5—Co1—N1—C2	−86.23 (17)	Co1—N2—C11—C10	−173.46 (18)
O1—Co1—N1—C2	4.96 (16)	C7—N2—C11—C12	−174.5 (2)
N2—Co1—N1—C2	−175.15 (17)	Co1—N2—C11—C12	11.6 (3)
N4—Co1—N1—C2	91.71 (17)	C9—C10—C11—N2	0.0 (4)
N3—Co1—N2—C11	11.2 (2)	C9—C10—C11—C12	175.1 (2)
N1—Co1—N2—C11	−178.3 (2)	N2—C11—C12—O9	77.1 (3)
O5—Co1—N2—C11	94.5 (2)	C10—C11—C12—O9	−98.1 (3)
O1—Co1—N2—C11	−177.8 (3)	N2—C11—C12—O10	−104.8 (3)
N4—Co1—N2—C11	−71.3 (2)	C10—C11—C12—O10	80.0 (3)
N3—Co1—N2—C7	−163.00 (15)	Co1—O5—C13—O6	−171.1 (2)
N1—Co1—N2—C7	7.52 (15)	Co1—O5—C13—C14	7.4 (2)
O5—Co1—N2—C7	−79.74 (16)	C18—N3—C14—C15	3.0 (3)
O1—Co1—N2—C7	8.0 (4)	Co1—N3—C14—C15	175.12 (17)
N4—Co1—N2—C7	114.45 (16)	C18—N3—C14—C13	−174.2 (2)
N1—Co1—N3—C14	−30.2 (5)	Co1—N3—C14—C13	−2.1 (2)
O5—Co1—N3—C14	4.85 (16)	O6—C13—C14—N3	175.0 (2)
O1—Co1—N3—C14	−85.95 (16)	O5—C13—C14—N3	−3.6 (3)
N2—Co1—N3—C14	91.80 (16)	O6—C13—C14—C15	−1.9 (4)
N4—Co1—N3—C14	−174.78 (17)	O5—C13—C14—C15	179.5 (2)
N1—Co1—N3—C18	142.1 (4)	N3—C14—C15—C16	−0.7 (3)
O5—Co1—N3—C18	177.15 (18)	C13—C14—C15—C16	176.0 (2)
O1—Co1—N3—C18	86.35 (18)	C14—C15—C16—C17	−0.9 (4)
N2—Co1—N3—C18	−95.90 (18)	C15—C16—C17—C18	0.2 (4)
N4—Co1—N3—C18	−2.48 (17)	C14—N3—C18—C17	−3.7 (3)
N3—Co1—N4—C23	178.3 (2)	Co1—N3—C18—C17	−175.46 (16)
N1—Co1—N4—C23	5.0 (2)	C14—N3—C18—C19	175.02 (19)
O5—Co1—N4—C23	176.9 (2)	Co1—N3—C18—C19	3.3 (3)
O1—Co1—N4—C23	87.71 (19)	C16—C17—C18—N3	2.0 (3)
N2—Co1—N4—C23	−78.42 (19)	C16—C17—C18—C19	−176.5 (2)
N3—Co1—N4—C19	0.99 (15)	C23—N4—C19—C20	2.2 (3)
N1—Co1—N4—C19	−172.30 (15)	Co1—N4—C19—C20	179.82 (18)
O5—Co1—N4—C19	−0.5 (4)	C23—N4—C19—C18	−177.24 (19)
O1—Co1—N4—C19	−89.61 (16)	Co1—N4—C19—C18	0.4 (2)
N2—Co1—N4—C19	104.26 (16)	N3—C18—C19—N4	−2.2 (3)
Co1—O1—C1—O2	−177.4 (2)	C17—C18—C19—N4	176.4 (2)
Co1—O1—C1—C2	1.1 (2)	N3—C18—C19—C20	178.4 (2)
C6—N1—C2—C3	4.3 (3)	C17—C18—C19—C20	−3.0 (4)
Co1—N1—C2—C3	169.50 (18)	N4—C19—C20—C21	−0.9 (4)
C6—N1—C2—C1	−170.7 (2)	C18—C19—C20—C21	178.4 (2)
Co1—N1—C2—C1	−5.5 (2)	C19—C20—C21—C22	−1.0 (3)
O2—C1—C2—N1	−178.7 (2)	C20—C21—C22—C23	1.8 (4)
O1—C1—C2—N1	2.8 (3)	C19—N4—C23—C22	−1.4 (3)
O2—C1—C2—C3	6.8 (4)	Co1—N4—C23—C22	−178.62 (18)
O1—C1—C2—C3	−171.7 (2)	C19—N4—C23—C24	178.1 (2)
N1—C2—C3—C4	−1.5 (4)	Co1—N4—C23—C24	0.9 (3)
C1—C2—C3—C4	172.5 (2)	C21—C22—C23—N4	−0.5 (4)
C2—C3—C4—C5	−2.0 (4)	C21—C22—C23—C24	180.0 (2)
C3—C4—C5—C6	2.8 (4)	N4—C23—C24—O8	82.6 (3)
C2—N1—C6—C5	−3.5 (3)	C22—C23—C24—O8	−97.9 (3)
Co1—N1—C6—C5	−168.27 (18)	N4—C23—C24—O7	−99.5 (3)
C2—N1—C6—C7	173.7 (2)	C22—C23—C24—O7	80.1 (3)

### Hydrogen-bond geometry (Å, º)

**Table:**

*D*—H···*A*	*D*—H	H···*A*	*D*···*A*	*D*—H···*A*
O10—H10*A*···O7^i^	0.82	1.65	2.445 (3)	164
C15—H15···O2^i^	0.93	2.58	3.391 (3)	147
C8—H8···O10^ii^	0.93	2.41	3.142 (3)	135
C16—H16···O6^iii^	0.93	2.52	3.044 (3)	116
C20—H20···O1^iv^	0.93	2.55	3.315 (3)	140
C22—H22···O9^v^	0.93	2.35	3.091 (3)	136

Symmetry codes: (i) *x*, −*y*+3/2, *z*−1/2; (ii) −*x*+1, *y*+1/2, −*z*+3/2; (iii) −*x*+2, *y*−1/2, −*z*+3/2; (iv) −*x*+2, −*y*+1, −*z*+2; (v) −*x*+1, −*y*+1, −*z*+2.
